# Evaluating UAV-Based Remote Sensing for Hay Yield Estimation

**DOI:** 10.3390/s24165326

**Published:** 2024-08-17

**Authors:** Kyuho Lee, Kenneth A. Sudduth, Jianfeng Zhou

**Affiliations:** 1 Department of Chemical and Biomedical Engineering, University of Missouri, Columbia, MO 65211, USA; elkyu0927@cnu.ac.kr; 2 Department of Smart Agricultural System, Graduate School, Chungnam National University, Daejeon 34134, Republic of Korea; 3 Department of Agricultural Machinery Engineering, Graduate School, Chungnam National University, Daejeon 34134, Republic of Korea; 4 USDA-ARS Cropping Systems and Water Quality Research Unit, Columbia, MO 65211, USA; 5 Division of Plant Science and Technology, University of Missouri, Columbia, MO 65211, USA; zhoujianf@missouri.edu

**Keywords:** hay yield-monitoring system, multispectral image, precision agriculture, remote-sensing technology, UAV

## Abstract

(1) Background: Yield-monitoring systems are widely used in grain crops but are less advanced for hay and forage. Current commercial systems are generally limited to weighing individual bales, limiting the spatial resolution of maps of hay yield. This study evaluated an Uncrewed Aerial Vehicle (UAV)-based imaging system to estimate hay yield. (2) Methods: Data were collected from three 0.4 ha plots and a 35 ha hay field of red clover and timothy grass in September 2020. A multispectral camera on the UAV captured images at 30 m (20 mm pixel^−1^) and 50 m (35 mm pixel^−1^) heights. Eleven Vegetation Indices (VIs) and five texture features were calculated from the images to estimate biomass yield. Multivariate regression models (VIs and texture features vs. biomass) were evaluated. (3) Results: Model R^2^ values ranged from 0.31 to 0.68. (4) Conclusions: Despite strong correlations between standard VIs and biomass, challenges such as variable image resolution and clarity affected accuracy. Further research is needed before UAV-based yield estimation can provide accurate, high-resolution hay yield maps.

## 1. Introduction

Precision agriculture (PA) represents a significant advancement in agriculture by integrating advanced technologies with data-driven approaches. PA uses multiple data sources, including temporal, spatial, and crop-specific information, to enhance farming operations. Recognizing variations in soil properties, nutrient levels, and environmental factors allows for the site-specific management of fertilizers, water, and pesticides. This targeted input approach aims to improve resource efficiency, productivity, product quality, profitability, and sustainability [1]. Historically, site-specific management faced challenges due to the spatial precision required in data collection and field management complexity. The integration of Global Navigation Satellite System (GNSS) technologies significantly advanced these efforts by enabling precise georeferenced spatiotemporal data collection [2].

Hay plays a key role in livestock health and productivity, especially during periods with limited pasture, like winter or drought, providing essential nutrients and energy [3]. Hay production helps manage the risks from climate variability and supports sustainable agriculture. Choosing grass and legume varieties suited to specific regions enhances ecosystem resilience [4,5]. Perennial hay crops adapt well to different conditions, improving soil health and reducing erosion. Varieties like Timothy, Alfalfa, Orchardgrass, and Bermuda grass meet livestock dietary needs and provide flexible feeding options [6,7]. Hay sales significantly impact rural economies, but they are influenced by factors like weather, feed demand, and trade policies [8].

Accurate yield-monitoring systems enhance productivity and efficiency in crop production. These systems provide essential data for strategic decisions in harvesting, such as optimal scheduling and resource utilization. Yield data also help refine agronomic practices like irrigation, fertilization, and pest management [9]. Traditionally, hay yield monitoring has relied on counting and weighing bales in the field, providing accurate but not site-specific data. One study found an R^2^ of 0.98 when comparing manually measured and sensor-measured bale weights using a chute transducer at 10 km h^−1^ [10]. However, precision agriculture requires higher spatial resolution for accurate management. To enhance accuracy and provide site-specific information, proximal sensors like ultrasonic and LiDAR, as well as remote sensing from satellites, crewed aircraft, or UAVs, can be utilized.

In agriculture, commonly used remote sensors include visible cameras, and multispectral and hyperspectral imagers, which measure reflected and emitted radiation to quantify physical characteristics. Time-of-flight (ToF) sensors detect plant height and dimensions. Remote sensing can efficiently monitor biomass in hay and forage crops across large fields quickly. While satellite imagery is useful for regional vegetation monitoring, its limitations include low resolution, high image costs, and infrequent acquisition schedules. Advances in UAV technology and image processing have made UAV imaging systems popular for their high resolution, controlled acquisition, and relatively low cost.

Most previous research on biomass estimation involved imaging a single species (e.g., soybean or tall fescue). However, a few studies estimated biomass in a mixed-species scene with moderate success. For example, the biomass of mixed grass was estimated using a UAV-mounted RGB camera, finding an R^2^ value of 0.6 between UAV-derived plant height and dry biomass at a 60 m flight altitude; however, the inclusion of RGBVI reduced the R^2^ to 0.5 [11]. Similarly, other research used UAV RGB imaging to collect data from mixed grasslands, achieving R^2^ values between 0.36 and 0.65 for dry weight estimation, with the normalized green–red difference index (NGRDI) as a significant predictor [12].

Low-cost UAVs with multispectral and RGB cameras were employed to study timothy grass [13]. Linear and multiple regression models based on image data showed greater accuracy (R^2^ = 0.48–0.72) than using LiDAR-measured plant height for biomass estimation. Another study predicted alfalfa yields and nutritional values using UAV imagery of canopy height and various crop parameters, identifying the Gaussian random process model (GRP) as the best, with R^2^ values between 0.78 and 0.81 [14].

In previous studies on biomass estimation through remote sensing, multiple image acquisitions throughout the growing season were frequently employed. For instance, weekly or bi-weekly data collection across 200 plots, each 45 m² in size, was performed to create training and test datasets [15]. While this method can yield more accurate results, it is likely impractical for commercial yield mapping. Using a single imaging date at harvest time would require less extensive ground truth-data collection and would be more appealing to producers. The goal of this research was to provide an efficient alternative to the conventional methods that rely on bale weights for estimating hay yield, thereby improving the spatial resolution of hay yield data. While estimating hay yield through bale weighing is effective for practical, non-spatial applications, it does not provide the spatially detailed hay yield information required for site-specific management. The specific objectives were (1) to develop an efficient methodology using multispectral remote-sensing images for predicting hay yield and (2) to assess the effectiveness of various VIs and texture features in multivariable regression models.

## 2. Materials and Methods

### 2.1. Field Information

**Figure 1:**
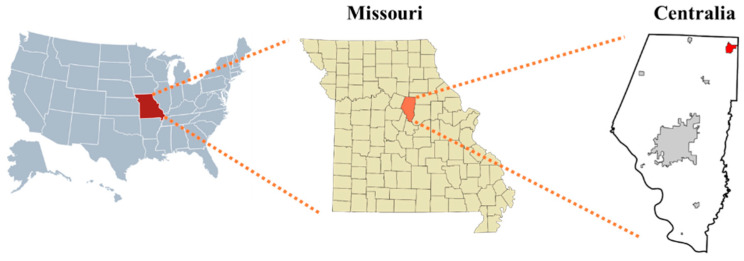
Field location: cropping-system experiment in Central Missouri, near Centralia (39°13 N, 92°07 W).

The data-collection site for this research is located 2 km from Centralia, Missouri (39°13 N, 92°07 W; Figure 1). The 35 ha research field and 30 adjacent 0.4 ha plots were established in 1991 by the USDA-ARS Cropping Systems and Water Quality Research Unit and the University of Missouri as part of a multi-state research project.

**Figure 2:**
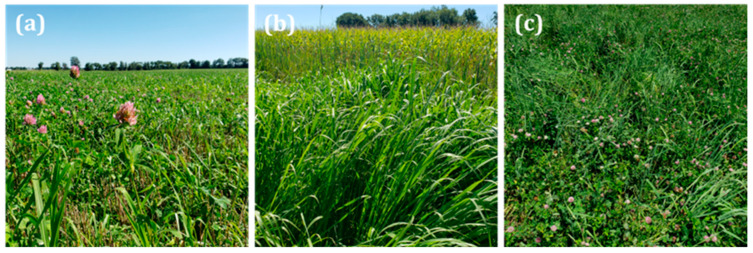
Different proportions of timothy grass and red clover were found in different areas of the experimental field: (**a**) mainly red clover, (**b**) mainly timothy grass, and (**c**) mixture of timothy grass and red clover.

In the last decade, this site has become part of the Central Mississippi River Basin (CMRB) location in the USDA’s Long-Term Agroecosystem Research (LTAR) network [16]. The LTAR research design includes a comparison of an “aspirational” (ASP) cropping system designed to improve environmental and economic sustainability with a “business as usual” (BAU) system commonly used by farmers in the area. The ASP system, which consists of a corn–soybean–wheat–hay rotation, has been implemented on the field and three of the adjacent plots. Production is rainfed, following standard management practices. In March 2020, the field and three adjacent plots were seeded with a mixture of red clover (Trifolium pratense L; 5.6 kg ha^−1^) and timothy grass (Phleum pratense; 11.2 kg ha^−1^). The seed was mixed with fertilizer and applied to the existing wheat crop using an air-boom applicator so that the hay could be well-established by the time of wheat harvest. Due to non-uniform plant establishment, some areas of the field were dominated by timothy, while others were predominantly red clover (Figure 2). To account for this variation, the proportion of grass and clover in the hay was visually rated at each sampling location using a 1–5 scale (Table 1).

### 2.2. Ground Truth-Data Collection

Data collection included collecting ground-truth biomass data at selected sample locations and UAV-based remote-sensing data collection for the three hay plots and the field. All field data were collected at the maturity stage (i.e., reproductive and flowering) of hay on 14 and 15 September 2020, one day before and on the day of harvest. Harvesting at the reproductive or flowering stage has been reported to be the best time to obtain a high grass or legume yield [17].

**Figure 3:**
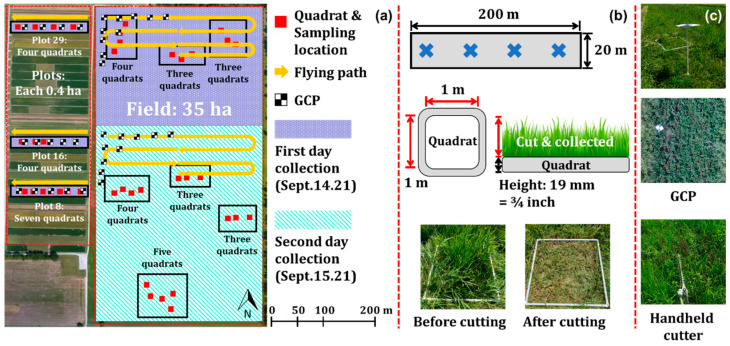
Experimental information on plots and field: (**a**) quadrat (sampling) locations, GCP locations, and flight path; (**b**) plot and quadrat dimensions, and inside of the quadrats before and after sampling; and (**c**) GCP on the field and handheld cutter images.

Before sensing data were collected, sampling sites were established in both the plots and large field using 1 m × 1 m quadrats made of PVC pipes with a diameter of 19 mm (Figure 3a). Fifteen quadrats were placed on the plots (seven on plot 8, four on plot 16, and four on plot 29), and twenty-five quadrats were placed on the large field. On the field, data were collected over two days due to the battery limitations of the UAV. The northern part of the field was sampled on 14 September, and the southern part was sampled on 15 September. In Figure 3a, the purple area was sampled on the first day, and the light green part was completed on the second day. The location of each quadrat was measured using a RTK GNSS system (Reach RS+, Emlid, Saint Petersburg, Russia) at the center of the quadrat. Also, the coordinates of Ground Control Points (GCPs) were determined by RTK-GNSS for comparison with their position in the UAV images (Figure 3c).

The height of the mixed vegetation inside the quadrats was measured at three locations, using a tape measure with 10 mm precision. All vegetation within the quadrat was cut at approximately 20 mm above the ground, using a handheld grass cutter (STIHL FS 90R, STIHL Corporation, Waiblingen, Germany), after the sensing data were collected (Figure 3c). All vegetation from each quadrat was collected into a paper bag that was sealed to reduce moisture loss and labeled properly.

The wet mass (g) of the harvested hay from each sampling location was measured on the day of collection, using a digital scale. A subsample from each harvested sample was weighed and dried at 105 °C for 24 h, using a laboratory oven (SHEL LAB SMO28-2, Sheldon Manufacturing Inc., Cornelius, OR, USA) to determine the subsample dry mass (g) using standard methods [18]. The dry-basis moisture content percentage (MC_db_) and dry mass of the overall samples were then calculated using Equations (1) and (2). Dry-basis moisture content (i.e., when the denominator of Equation (1) is dry mass) can be greater than 100% for wetter samples. We chose the dry-basis metric because it has the advantage that a change in dry-basis moisture is linearly related to the weight loss or gain of the sample. The more common wet-basis moisture content (MC_wb_) can be calculated from the MC_db_ by Equation (3).

MC_db_ (%) = (Subsample wet mass-Subsample dry mass)/(Subsample dry mass) × 100 (1)

Sample dry mass (g) = (Subsample dry mass)/(Subsample wet mass) × Sample wet mass(2)

MC_wb_ (%) = (MC_db_/(100 + MC_db_)) × 100 (3)

### 2.3. UAV System and Remote Sensing Data Collection

A multispectral imaging system consisting of a multispectral camera (MicaSense: RedEdge-M, Seattle, WA, USA) attached to a UAV platform (DJI Matrice 600 Pro, DJI, Shengzhen, Guangdong, China) was used to acquire images at 30 m above ground level (AGL) for the plots and 50 m AGL for the field, resulting in a ground sample distance (GSD) of 20 and 34 mm pixel^−1^, respectively. Details of data acquisition are given in Table 2. The multispectral camera consisted of five independent single-band cameras (red, green, blue, red edge, and near-infrared) and was configured to take time-lapse images at 1 frame per second (fps) using UAV control software (Autopilot v4.7.1, Hangar Technology, Austin, TX, USA) installed on an iPad mini 4 (Apple Inc., Cupertino, CA, USA). Before and after each flight, a calibration reflectance panel (CRP) provided by the company was imaged by holding the camera at about 1.0 m above the CRP and looking down vertically in an open area to avoid shadows [19].

Images were acquired from 10 am to 1 pm Central Daylight Time on 14 September 2020 for the plots and on 15 September 2020 for the field (Table 2). Both days had a clear sky, with an occasional strong wind. Images were taken at 30 and 50 m above ground level (AGL) at a flight speed of 7 km h^−1^ following a zigzag path to cover the plots and field with forward overlap ≥70% and side overlap ≥65%.

### 2.4. Data Processing and Analysis

**Figure 4:**
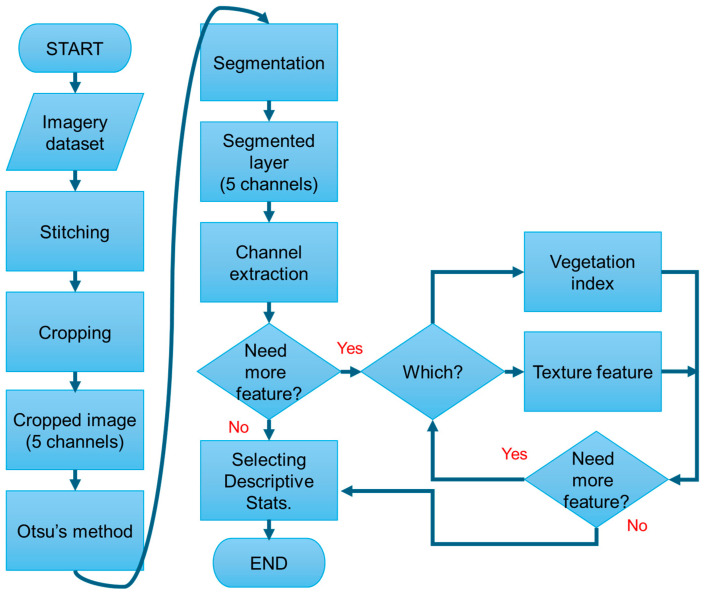
Image pre-processing from acquisition to feature selection.

Figure 4 shows the specific image pre-processing procedure used in the study. The first step was to stitch the multispectral images with Pix4Dmapper with Ag Multispectral template (Pix4D, Prilly, Switzerland) to create orthomosaic images and digital elevation models (DEM) for further processing. All quadrats were visually identified and cropped from the five bands of the orthomosaic images, and those cropped images were used to calculate the values of the vegetation indices (VIs) for each quadrat using MATLAB (R2023a, MathWorks, Natick, MA, USA). The background of the cropped images, including soil, crop residues, and other non-crop (non-green) material, was removed using Otsu’s method [20]. Eleven image features (VIs) were calculated from the images (Table A1). These VIs were selected to discriminate the hay crop and avoid soil, shadow, and light effects within the quadrat images. Statistics, including Mean, Median, Quantile 10%, Quantile 90%, Maximum, and Minimum values, of the eleven VIs were calculated for each quadrat image for use in biomass estimation.

Image texture features, which distinguish objects based on their shape or pattern, and are widely used in image analysis, were also investigated. For example, even if the spectral composition of a biomass image did not change, image texture features might be able to discriminate among biomass levels. In this research, contrast, correlation, energy, entropy, and homogeneity of the gray-level co-occurrence matrix (GLCM), which gives the spatial relationship between adjacent or neighboring pixels, were used to describe the texture of the images inside of the quadrats (Table A2) [21].

After all procedures for pre-processing were completed, the next step was to find the optimal subset of predictor variables in order to reduce the effect of noise or uncorrelated variables, decrease the runtime of modeling, and improve the prediction performance. In this study, Recursive Feature Elimination (RFE) and Pearson’s correlation coefficient were used to select important image features for modeling. The RFE method evaluates and selects features for a machine-learning model according to their impact on its performance [22]. This approach identifies highly correlated variables, ranking features based on their correlation, both among independent variables and between independent and dependent variables. The lower-ranked features are systematically removed. In the case of image and texture-feature selection, the RFE algorithm identifies key variables, which are then used to construct models. Furthermore, a correlation analysis is performed to examine the linear relationships among these variables, aiming to enhance model efficiency by integrating RFE with Pearson’s correlation coefficient for more effective variable selection.

A multiple linear regression model was employed to predict hay biomass using extracted image features, including VIs and texture features. The independent variables included eleven VIs and five texture features derived from the multispectral images. The dependent variable was the biomass yield measured in the field. The regression model used in this study is expressed in Equation (4):

(4) Y=β0+β1⋅VI1+β2⋅VI2+…+β11⋅VI11+β12⋅T1+…+β16⋅T5+ϵ

where Y = hay biomass (g); β0 = intercept; β1, β2, …, β11 = regression coefficients associated with VIs; VI1, VI2,…VI11 = values of the Vis; β12, …,β16 = regression coefficients associated with texture features; T1, …,T5 = values of the texture features derived from image analysis; and ϵ = error term.

A10-fold cross-validation resampling method was utilized to avoid overfitting. This approach involved dividing the entire dataset into ten subsets, with each subset being used once as a test set, while the remaining subsets formed the training set. This method was chosen to enhance the robustness of the model by ensuring that all data points contributed to both training and validation. The model’s performance was evaluated based on R^2^, RMSE, and MAE values. RStudio (version 4.4; RStudio, Boston, MA, USA) with the “caret” package (version 6.0-80) was used for the analysis.

## 3. Results

### 3.1. Ground-Truth Data: Comparison and Mass

The height of quadrat biomass was variable, with a maximum of 1.3 m and an average height of 0.69 m in the plots and 0.73 m in the field. The average wet mass of samples was calculated to be 0.9 kg, and the average dry mass was 0.4 kg. A total of eight groups were created by the variation in the composition of the samples inside the quadrats (Table 3). In addition, Table 4 shows the distribution of the sample dry weight. Even if the moisture content at some points was affected by wheat straw and weeds such as panicum, foxtail, and waterhemp, there was an approximately normal distribution of dry mass concentrated in the range from 0.3 to 0.4 kg.

According to Table 3, most of the plot and field sampling locations were dominated by timothy grass. The dry mass was larger in the quadrats containing more red clover than grass. A few samples included a noticeable amount of non-hay material, such as wheat straw and weeds such as foxtail, panicum, and water hemp. To assess the effect of these materials on the wet and dry sample mass, two ANOVA tests were conducted. These tests compared wet and dry mass between the “Grass and clover” and “Including other biomass” groups (the latter containing non-hay materials), as detailed in Table 3. The ANOVA results indicated that the dry mass of the samples was not significantly affected by these non-hay materials, as evidenced by a *p*-value greater than 0.05. In contrast, the wet mass showed a significant difference, with *p*-values of 0.018, which is less than the 0.05 threshold for statistical significance. This suggests that while the non-hay materials and the proportions of grass versus clover do not impact the dry mass, they significantly affect the wet mass of the samples.

### 3.2. Model Performance

#### 3.2.1. VI Correlation

**Figure 5:**
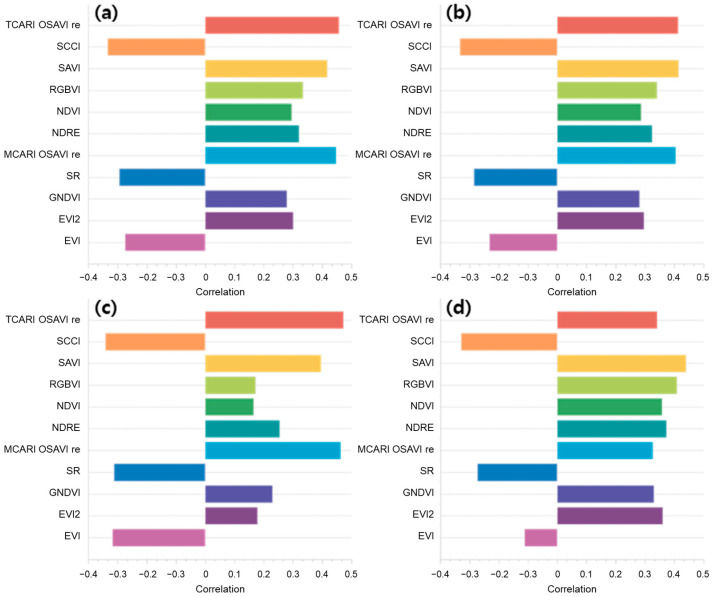
Pearson correlation coefficients between VIs and dry mass: (**a**) Mean, (**b**) Median, (**c**) Quantile 90%, and (**d**) Quantile 10%.

Pearson correlations between VIs and dry mass are shown in Figure 5. Because of very low correlations observed with maximum and minimum values (i.e., −0.1 < r < 0.1), those results were not included. According to the graph, SR, EVI, and SCCCI (see Appendix A for definitions) had a negative relationship with dry mass. This could indicate that these indices are sensitive to factors that inversely affect vegetation dry mass, possibly due to their design. Correlations of SAVI, TCARI/OSAVI red edge, and MCARI/OSAVI red edge (Appendix A) with dry mass were generally among the highest obtained for the VIs tested.

#### 3.2.2. RFE Results

**Figure 6:**
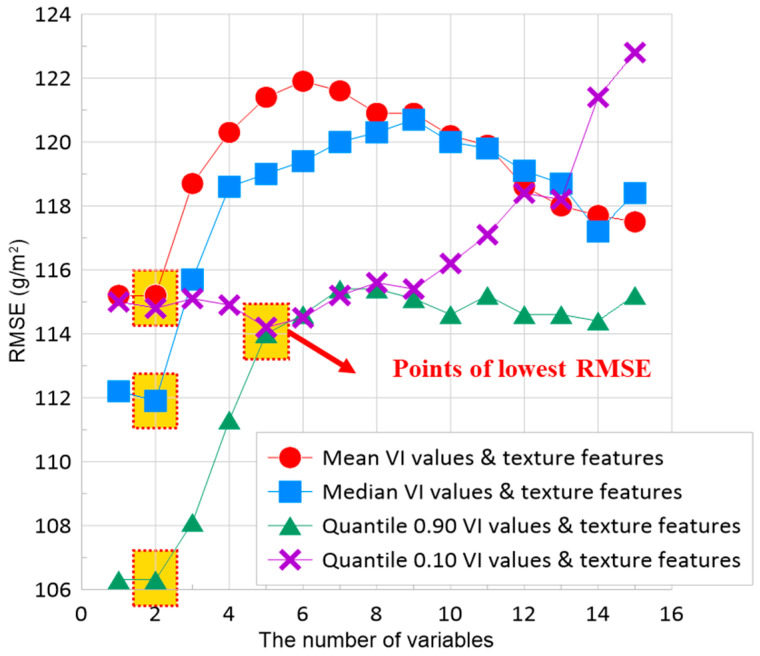
Optimum number of variables indicated by RFE using ten-fold cross validation. Dashed squares indicate the lowest RMSE and the corresponding recommended number of variables. The optimal number of independent variables was two for texture features and Mean, Median, and Quantile 90% summarization of VI values; and it was five for texture features, and Quantile 10% summarization of VI values.

Figure 6 shows the results of the RFE analysis, including the optimal number of variables for regression models and the RMSE of the RFE analysis. The RFE analysis included the eleven VIs and the five texture features as independent variables. Two variables were recommended for models using texture features plus the Mean, Median, or Quantile 90% summarization of VIs, and five variables were recommended for models using texture features plus the Quantile 10% summarization of VIs (Figure 6).

Table 5 shows the RFE model results for two sets of independent variables, VIs plus texture features (Figure 6) and VIs alone. The optimum variables were the same whether texture features were included or not: (1) MCARI/OSAVI red edge and TCARI/OSAVI red edge when two variables were optimal; and (2) MCARI/OSAVI red edge, NDRE, RGBVI, SAVI, and TCARI/OSAVI red edge when five variables were optimal. Evidently, texture features were less important for predicting biomass than VIs and therefore were not recommended by the RFE algorithm. To further assess the importance of texture features, a separate RFE was conducted with texture features alone.

The RFE results in Table 6 recommend that all five texture variables be used in texture-only modeling. However, this raises concerns about model overfitting. Because of this, and in the interest of model parsimony, a single texture feature, energy, was selected based on Pearson’s correlation values.

#### 3.2.3. Model Results Based on RFE

The results of the multiple linear regression models using only VI variables and models using VIs and texture-feature variables are summarized in Table 7. Models were developed separately for the plots and the field, as well as for the plots and field combined. In general, adding the energy texture feature to the VI models increased the R^2^ and decreased the RMSE and MAE, showing that adding the texture feature could improve model accuracy. Overall, prediction accuracy for plots alone was higher than for the field or plots and field. Selecting a Quantile 10% summarization of VI data was the best choice to estimate dry mass in the study (Table 7).

**Figure 7:**
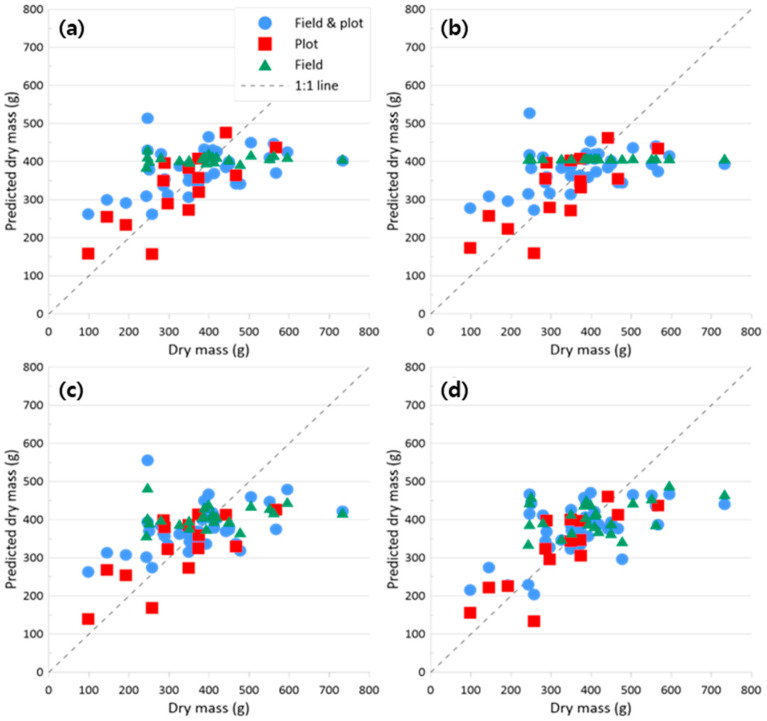
Scatter plots between predicted dry mass and measured dry mass with models using the RFE results of VIs only: (**a**) Mean, (**b**) Median, (**c**) Quantile 90%, and (**d**) Quantile 10%.

**Figure 8:**
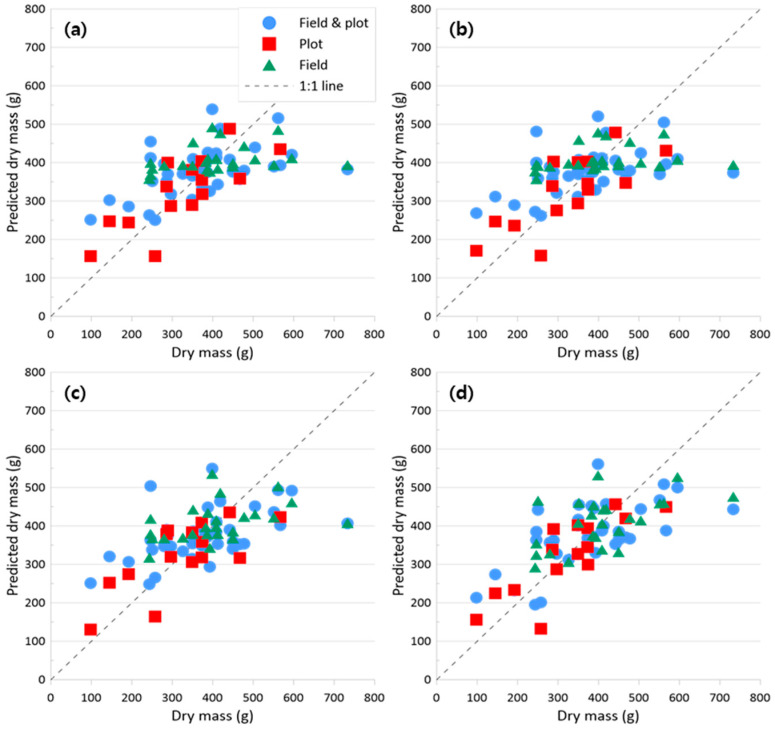
Scatter plots between predicted dry mass and measured dry mass with models using the RFE results of VIs (MCARI OSAVI red edge, NDRE, RGBVI, SAVI, and TCARI OSAVI red edge) and the energy texture feature: (**a**) Mean, (**b**) Median, (**c**) Quantile 90%, and (**d**) Quantile 10%.

Figure 7 shows the relationship between predicted and measured dry mass for models using only VIs, while Figure 8 shows the relationship when VIs and the energy texture feature were included. When using VIs alone (Figure 7), Quantile 10% summarization provided the best plot results (R^2^ = 0.68, RMSE = 17.84%, and MAE = 54.49 g m^−2^). Quantile 10% results also provided the best results in the VI-plus-texture-feature model (Figure 8). However, the results were only slightly improved with the addition of the energy texture feature (R^2^ = 0.68, RMSE = 17.72%, and MAE = 56.44 g m^−2^).

## 4. Discussion

Previous research indicates that estimating yield using proximal-sensing, remote-sensing, or combined sensing approaches holds significant potential for providing spatial information. Nonetheless, there are limitations to both proximal- and remote-sensing systems in estimating hay yield or biomass, as summarized in Table 8. Common issues include low estimation accuracy, particularly in fields with mixed plant species, low crop density, or heterogeneous conditions such as varying grazing intensities, which affect biomass levels, plant structures, and spectral characteristics [23]. Additional factors impacting accuracy include selecting optimal data-collection dates; environmental conditions, such as wind, dust, and dirt; and the condition of the crops being sensed [24,25,26,27].

### 4.1. Ground-Truth Data

Several reasons for the relatively low accuracy of the remote-sensing models developed in this study were considered. One of these could be the variation in the plant material present across the different sampling locations. Twelve out of the forty quadrat samples included a large amount of wheat straw, panicum, foxtail, and/or waterhemp, with ten of these found in the field (Table 5). The presence of these plant materials other than red clover and timothy grass could affect the dry mass of the samples because wheat straw would reduce the samples’ moisture content, while weeds such as waterhemp would increase the samples’ moisture content. However, since there were only forty quadrats, it was difficult to know how much biomass was influenced by the presence of straw and weeds and its effect on the independent variables obtained from the remote-sensing images.

### 4.2. Remote-Sensing Data

Blurry images and low resolution in the multispectral image data might be one reason for the low accuracy of the regression models. To investigate this issue, an RGB camera mounted on another UAV (DJI Phantom 4 Advanced, DJI, Shengzhen, Guangdong, China) was deployed in the plots to collect comparison data. Details for RGB data collection in the plots are summarized in Table 9. This UAV was flown at an altitude of 20 m AGL, achieving a GSD of 5 mm pixel^−1^, compared to the 20 mm pixel^−1^ GSD during the multispectral-data collection. The RGB camera captured images at 0.5 fps, controlled by the Litchi UAV application (VC Technology Ltd., London, UK).

**Figure 9:**
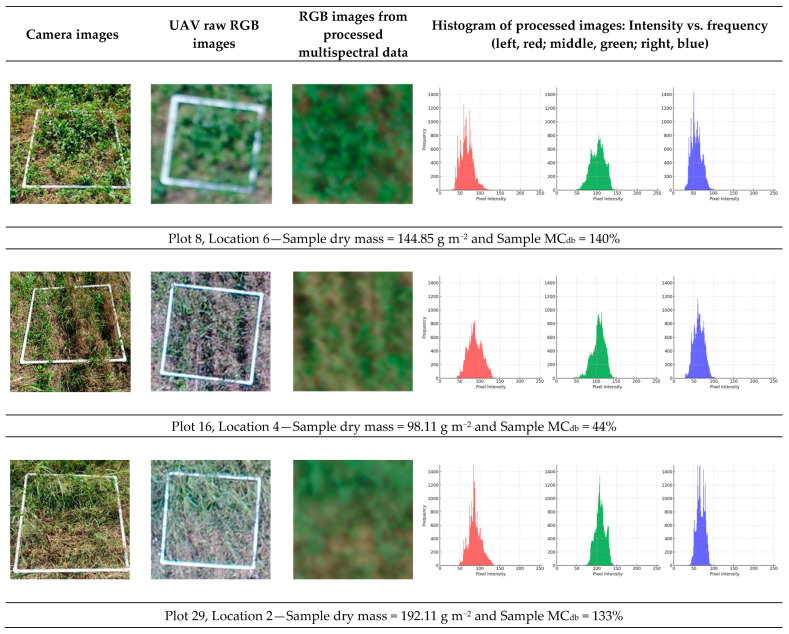
Images obtained from several locations in the plots using a commercial smartphone camera, RGB UAV images, and processed multispectral UAV images.

Figure 9 compares various image types at several locations within the plots. Images from a commercial smartphone camera, RGB images captured by the UAV-mounted RGB camera, and pre-segmentation processed images extracted from the multispectral image data are compared. The images in the third column are composed of RGB bands from the multispectral images, with the final column displaying intensity histograms for the red, green, and blue channels. The histograms might indicate that higher frequencies of green and blue correlated with increased dry mass (Plot 8, 144.85 g m^−2^; and Plot 29, 192.11 g m^−2^), while higher red frequencies suggested lower weight (Plot 16: 98.11 g m^−2^). The comparison shows that multispectral images were generally less clear than those from the RGB camera and the smartphone, which could have affected the performance of models using that data. When field images, which were affected by strong winds and/or lower resolution (due to higher AGL), were excluded, the multispectral image model performance could potentially improve to an R^2^ value of 0.52 to 0.68 (Table 7).

**Figure 10:**
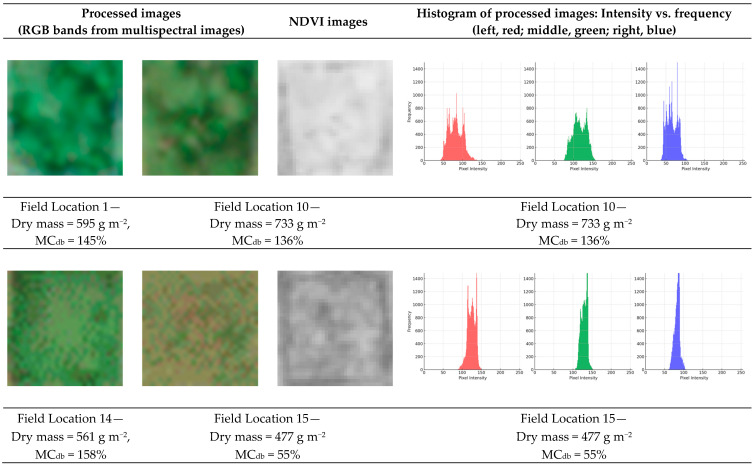
Images obtained from several locations in the field, including RGB-processed images, NDVI images, and histograms of the RGB images.

Figure 10 displays processed multispectral and NDVI images of field samples. Similar to Figure 9, the color images were derived from the RGB bands of multispectral image data. While images from Field Locations 1 and 10 were similar to those in Figure 9, those from Field Locations 14 and 15 exhibited resolution issues. Despite setting the UAV flight height at 20 m, resulting in an expected ground resolution of 5 mm pixel^−1^ for the RGB camera and 14 mm pixel^−1^ for the multispectral camera, actual measurements varied slightly. The altitude discrepancy was less significant for the RGB-equipped UAV (1-to-3 m difference) compared to the multispectral UAV (3-to-7 m difference). This variance in altitude was reflected in the image resolution, with the RGB camera showing a difference from 0 to 1 mm pixel^−1^, and the multispectral camera showing a difference from 0 to 2 mm pixel^−1^, though field discrepancies were larger (1 to 6 mm pixel^−1^). These differences in resolution might have contributed to the less accurate models obtained with the field data (R² values < 0.3). In addition, due to the generally high correlation between NDVI and biomass, NDVI images were generated for interpreting the results. However, they did not exhibit discernable differences by visual inspection. As shown in Figure 5 and Table 5, the correlation coefficients for NDVI were below 0.4, and the RFE algorithm did not identify NDVI as a significant independent variable. The issue of low resolution might be a factor contributing to the low R² values observed in the field.

Furthermore, a difference in wavelength intensity patterns was observed between the histograms of well-processed and poorly processed images (Figure 9 and Figure 10). Visual inspection revealed that the intensity patterns for the plot locations in Figure 9 and Field Location 10 in Figure 10 were approximately normally distributed. In contrast, the histogram for Field Location 15 in Figure 10 displayed instability, with several high points. These results suggest that non-uniform sampling locations may not be effective for predicting spatial hay yield.

### 4.3. Biomass Estimation in a Mixed Hay Crop

Although red clover and timothy grass were the species present in the hay crop in this study, the mixed-crop scene was further complicated by the presence of significant wheat stubble and several weed species. The proportions of the different species varied greatly from place to place within the field. With the limited number of ground-truth samples available, this variability made it difficult to develop and validate a model that accurately estimated biomass across the study area.

Other studies using remote sensing to quantify mixed-crop biomass have had similar difficulties. A study [28] using high-resolution satellite imagery to predict cover crop biomass across a range of mixtures seeded with different species found a low accuracy in biomass estimations (R^2^ = 0.25). Accuracy could be improved (R^2^ = 0.61) if the species or mixture was used as a predictor variable. This was possible in their model, as the species or mixture composition was uniform across each study plot. Our situation was more complex, as the composition varied continuously across the study area due to variability in soil and landscape characteristics and other growing conditions.

Another study [29] estimated the biomass of a mixed grass and clover forage crop using a handheld field spectrometer (350–2500 nm). A validation R^2^ of 0.78 was obtained, based a model developed with 157 calibration samples and 78 validation samples. Compared to our study, the improved performance could be attributed to (1) the additional spectral information available from a spectrometer as opposed to a RGB sensor, (2) the potential for less measurement noise with proximal versus remote sensing, and (3) the larger ground-truth dataset for model development and validation.

A comprehensive review [30] of various UAV-based studies that estimated aboveground biomass in grasslands concluded that capturing the characteristics of diverse crop mixtures using RGB and multispectral sensors was challenging. It was noted that the inherent complexity of mixed species stands limited the accuracy of prediction models. The variability in plant structure and density across different species was reported to require more advanced models to effectively assess biomass. Also, the inclusion of structural, along with spectral, data was seen as important for improving accuracy. The authors noted the importance of additional research to develop multi-year and multi-site datasets that could support more global model development.

A study [31] employing a UAV-mounted hyperspectral (450–800 nm) imaging system for grassland biomass estimation found that multivariate models applied to hyperspectral data were much more accurate than simple regressions on multispectral VIs such as NDVI. They also emphasized the importance of a large number of ground-truth points for robust calibration and validation.

These previous studies support the need for our additional research in remote sensing-based biomass estimation of mixed-species grasslands. Along with the research we report here, they also highlight the necessity for integrating advanced computational techniques and proximal sensing technologies that can accommodate the intricacies of mixed-species environments. Implementing such technologies could improve the accuracy and reliability of biomass assessments.

## 5. Conclusions

This study examined the effectiveness of remote-sensing techniques for hay yield estimation, specifically multispectral UAV imagery analyzed with multiple linear regression models. An additional goal of this preliminary test was to develop methods that could be used to improve yield estimation in following studies and future large-scale hay experiments. The research process involved extracting and analyzing various image features, notably color space components and vegetation indices (VIs), from UAV-captured images. Notable correlations were found between certain VIs and biomass; however, issues such as variable image resolution were found to affect accuracy. The regression models indicated that the methods had potential, providing good fits (R² = 0.52 to 0.68, RMSE = 17.72 to 21.46%, and MAE = 56.44 to 67.68 g/m²) despite facing environmental and technological constraints. Variability in plant material across sampling sites impacted the accuracy of biomass estimates from remote-sensing data, and poor image clarity and resolution in UAV multispectral data affected the accuracy of regression models for biomass estimation.

## Figures and Tables

**Table 1:** Five-level scale of the proportion of grass and clover in samples.

Rating	Proportion of Timothy Grass and Red Clover
1	Mostly timothy grass
2	Approximately 2:1 timothy to red clover
3	Approximately equal
4	Approximately 1:2 timothy to red clover
5	Mostly red clover

**Table 2:** Summary of data collection conditions and UAV settings used for data collection.

Group	Location	Date (2020)	Ground Sample Distance (m)	Expected Ground Resolution (mm pixel^−1^)	Image Resolution (pixels × pixels)	Flight Speed (km h^−1^)	Frames per Second (fps)
Plot	Plot 8	14 Sept.	30	20	1280 × 960	7	1
	Plot 16	14 Sept.					
	Plot 29	14 Sept.					
Field	North	14 Sept.	50	34	1280 × 960	7	1
	Middle	15 Sept.					
	South	15 Sept.					

**Table 3:** Classification based on the plant material inside the quadrats.

Group for ANOVA Test		Composition	Sample Proportion (%)	Number of Sampling Locations in Each Composition *					
				Plot 8	Plot 16	Plot 29	N	M	S
All	Grass and clover	Mostly timothy grass	20.0	3	2	2	1	-	-
		Approximately 2:1 timothy to red clover	17.5	-	1	-	2	2	2
		Equal	5.0	1	-	-	1	-	-
		Approximately 1:2 timothy to red clover	7.5	-	-	-	3	-	-
		Mostly red clover	20.0	3	-	1	3	1	-
	Including other biomasses **	Mostly grass and 2:1 grass to clover	25.0	-	1	1	-	7	1
		Equal	2.5	-	-	-	-	1	-
		Mostly clover and 1:2 grass to clover	2.5	-	-	-	-	1	-

* N, M, and S refer to the northern, middle, and southern parts of the field, respectively. ** Other biomass: wheat straw and weeds.

**Table 4:** Distribution of sample dry mass in the plots and the field.

Dry Mass (kg)	Number of Quadrat Locations
Over 0.5	5
0.4–0.5	7
0.3–0.4	16
0.2–0.3	9
0.1–0.2	2
Under 0.1	1

**Table 5:** Results from RFE analysis of two different variable groups: (1) VIs plus texture features and (2) VIs alone. Bold numbers indicate the optimum number of variables.

Number of Variables	RMSE (g m^−2^)								Recommended Significant Variables *
	Mean		Median		Quantile 90%		Quantile 10%		
	All	Only VI	All	Only VI	All	Only VI	All	Only VI	
1	115.2	116.1	112.2	112.4	106.3	106	115	121.6	-
2	115.2	114.8	111.9	112	106.3	106	114.8	118.6	TCARI/OSAVI _RE_
3	118.7	115.9	115.7	112.8	108.1	108	115.1	117.5	MCARI/OSAVI _RE_
4	120.3	117.9	118.6	114.1	111.3	109.9	114.9	117.8	-
5	121.4	119.6	119	116.5	114	111	114.2	116.7	-
6	121.9	121	119.4	118.7	114.6	112.2	114.5	116.8	TCARI/OSAVI _RE_
7	121.6	122.5	120	119.8	115.4	113.4	115.2	117.7	MCARI/OSAVI _RE_
8	120.9	122.2	120.3	120.9	115.4	113.1	115.6	117.2	SAVI
9	120.9	121.9	120.7	119.9	115.1	113.1	115.4	124.8	RGBVI
10	120.2	122.9	120	122.7	114.6	114.8	116.2	124.7	EVI2
Optimum number of variables	2	2	2	2	2	2	5	5	

* RE = red edge.

**Table 6:** The results of the RFE employing texture features (contrast, correlation, energy, entropy, and homogeneity).

Number of Variables	R^2^	RMSE (g m^−2^)	MAE (g m^−2^)	Optimum Number of Variables	Significant Variables
1	0.33	142.5	117.67	Five	Contrast Correlation Energy Entropy Homogeneity
2	0.34	137.1	113.43		
3	0.29	134.5	110.61		
4	0.31	128.5	104.78		
5	0.38	117.9	96.18		

**Table 7:** Model results using the model structure recommended by RFE: either two or five VIs (Table 5), both alone and in combination with a single texture feature (energy).

Group	Statistics (Variables from RFE)	R^2^		RMSE (%)		MAE (g m^−2^)	
		VIs	VIs and Texture Feature	VIs	VIs and Texture Feature	VIs	VIs and Texture Feature
Plot	Quantile 90% *	0.52	0.53	21.79	21.46	71.26	67.68
	Quantile 10% **	0.68	0.68	17.84	17.72	54.49	56.44
	Mean *	0.61	0.61	19.76	19.66	64.80	64.65
	Median *	0.58	0.59	20.42	20.25	67.23	67.18
Field	Quantile 90% *	0.05	0.18	30.06	28.01	84.43	81.71
	Quantile 10% **	0.12	0.31	28.90	25.71	84.35	77.53
	Mean *	0.01	0.09	30.79	29.50	85.67	83.61
	Median *	0.00	0.07	30.88	29.71	85.54	83.07
Plot and field	Quantile 90% *	0.21	0.28	29.20	27.97	82.02	79.31
	Quantile 10% **	0.32	0.41	27.08	25.34	77.73	76.68
	Mean *	0.20	0.25	29.46	28.45	82.76	80.93
	Median *	0.16	0.21	30.12	29.29	83.37	82.19

* Independent variables: TCARI/OSAVI red edge, MCARI/OSAVI red edge (VIs), and energy (texture feature). ** Independent variables: TCARI/OSAVI red edge, MCARI/OSAVI red edge, SAVI, RGBVI, NDRE (VIs), and energy (texture feature).

**Table 8:** Limitations reported for hay yield estimation using proximal and remote sensing.

Sensing Type	Crops and Purpose	Measurements and Accuracy	Limitations	Reference
Proximal and remote (ultrasonic sensors and hyperspectral sensors on UAV)	Grass (Lolio-Cynosuretum)—data fusion and estimation of yield	Plant height and hyperspectral reflectance—R^2^ = 0.8	Lower accuracy caused by too-long or too-short sward heights	[23]
			Extremely heterogeneous grasslands such as are typical for very leniently stocked pastures	
Proximal and remote (ultrasonic sensors and hyperspectral sensors on UAV)	Grass (Lolio-Cynosuretum)—data fusion and estimation of yield	Plant height and hyperspectral reflectance—R^2^ = 0.5	Highly complex variation in pasture or sward structure	[26]
Proximal (infrared and ultrasonic distance sensors)	Mixed grass, bermudagrass, hybrid pearl millet, and oats—estimating plant height and yield	Plant height—15 to 20% yield prediction error	Tolerance to withstand dust and dirt	[25]
Proximal (ultrasonic sensors)	Grass—improving the accuracy of measuring plant height	Plant height—R^2^ = 0.8	Tilting or bouncing of sensor array	[27]
Remote (RGB camera on UAV)	Barley—simple estimation of yield	Plant height (crop surface model) and RGB reflectance—R^2^ = 0.8	Inability to collect data at specific growth stages due to weather (e.g., clouds and rain)	[24]

**Table 9:** Specifications of the RGB images collected in plots.

Ground Sample Distance (m)	Expected Ground Resolution (mm pixel^−1^)	Image Resolution (pixels × pixels)	Flight Speed (km h^−1^)	Frame per Second (fps)	Control Application
20	5	4864 × 3648	7	0.5	Litchi

## Data Availability

The data presented in this study are available upon request from the corresponding author, as they are being used in an ongoing study.

## Appendix A

**Table A1:** Vegetative indices investigated as potential predictors of hay yield.

Index	Equation *	Features	Reference
Enhanced Vegetation Index (EVI)	$\frac{2.5\times (NIR-R)}{\left(NIR+6\times R-7.5\times B+1\right)}$	- Correct NDVI results for air and soil influence - Appropriate for dense canopy area	[32]
Enhanced Vegetation Index 2 (EVI 2)	$\frac{2.5\times (NIR-R)}{\left(NIR+2.4\times R+1\right)}$	- Correct EVI results which is calculated by red and NIR bands	[33]
Green Normalized Difference Vegetation Index (GNDVI)	$\frac{\left(NIR-G\right)}{\left(NIR+G\right)}$	- More sensitive to chlorophyll concentration than NDVI	[34]
Simple Ratio (SR)	$\frac{NIR}{R}$	- Simple index to distinguish vegetation from soil	[35]
Modified Chlorophyll Absorption Ratio Index/Optimized Soil-Adjusted Vegetation Index red edge (MCARI/OSAVI red edge) **	$\mathrm{MCARI}\;\mathrm{red}\;\mathrm{edge}=[\left(NIR-RE\right)-0.2\times \left(NIR-G\right)]\times \frac{NIR}{RE}$	- Minimize the effect of non-photosynthetic materials on spectral estimates of absorbed photosynthetically active radiation	[36]
	$\mathrm{OSAVI}\;\mathrm{red}\;\mathrm{edge}=(1+0.16)\times \frac{\left(NIR-RE\right)}{\left(NIR+RE+0.16\right)}$	- Enhanced SAVI to minimize soil reflectance	
Normalized-Difference Red Edge Index (NDRE)	$\frac{\left(NIR-RE\right)}{\left(NIR+RE\right)}$	- Sensitive index to chlorophyll - Improve variation detection	[37]
Normalized Difference Vegetation Index (NDVI)	$\frac{\left(NIR-R\right)}{\left(NIR+R\right)}$	- Widespread index related to chlorophyll	[38]
Red–Green–Blue Vegetation Index (RGBVI)	$\frac{\left(G-B\times R\right)}{\left(G^{2}+B\times R\right)}$	- Related to chlorophyll - Enhanced index for green reflectance	[39]
Soil Adjusted Vegetation Index (SAVI)	$\frac{\left(1+0.5)\times (NIR-R\right)}{\left(NIR+R+0.5\right)}$	- Minimize soil brightness influence - Appropriate for arid regions	[35]
Simplified Canopy Chlorophyll Content Index (SCCCI)	$\frac{NDRE}{NDVI}$	- Sensitive to N uptake and total plant N% - Appropriate to recognize growth stage	[40]
Transformed Chlorophyll Absorption in the Reflectance Index/Optimized Soil-Adjusted Vegetation Index red edge (TCARI/OSAVI red edge) ***	$\mathrm{TCARI}\;\mathrm{red}\;\mathrm{edge}=3\times [\left(NIR-RE\right)-0.2\times (NIR-G)]\times \frac{NIR}{RE}$	- Indicate the relative abundance of chlorophyll - Affected by underlying soil reflectance and leaf area index	[36]
	$\mathrm{OSAVI}\;\mathrm{red}\;\mathrm{edge}=(1+0.16)\times \frac{\left(NIR-RE\right)}{\left(NIR+RE+0.16\right)}$	- Enhanced SAVI to minimize soil reflectance	

* B, G, NIR, R, and RE represent blue, green, near infrared, red, and red edge bands, respectively; ** MCARI/OSAVI Red edge = MCARI _Red edge_ ÷ OSAVI _Red edge_; *** TCARI/OSAVI Red edge = TCARI _Red edge_ ÷ OSAVI _Red edge_.

**Table A2:** Texture features from the GLCM [36] investigated as potential predictors of hay yield.

Index	Equation *	Features
Contrast	$\sum_{i=0}^{N_{g-1}}\sum_{j=0}^{N_{g-1}}\left({i-j)}^{2}\cdot g^{2}(i,j\right)$	- Measures the drastic change in gray level between contiguous pixels - High contrast images feature high spatial frequencies
Correlation	$\sum_{i=0}^{N_{g-1}}\sum_{j=0}^{N_{g-1}}\left({i-j)}^{2}\cdot g^{2}(i,j\right)$	- Measures the linear dependency in the image - High correlation values imply a linear relationship between the gray levels of adjacent pixel pairs
Energy	$\sqrt{\sum_{i=0}^{N_{g-1}}\sum_{j=0}^{N_{g-1}}g^{2}(i,j)}$	- Measures texture uniformity or pixel pair repetitions - High energy occurs when the distribution of gray level values is constant or periodic
Entropy	$\sum_{i=0}^{N_{g-1}}\sum_{j=0}^{N_{g-1}}g^{2}(i,j)\cdot \log\left(g\left(i,j\right)\right)$	- Measures the disorder of an image and is negatively correlated with Energy - Entropy is high when the image is texturally complex or includes much noise
Homogeneity	$\sum_{i=0}^{N_{g-1}}\sum_{j=0}^{N_{g-1}}\frac{1}{1+{\left(i-j\right)}^{2}}\cdot g(i,j)$	- Measures image homogeneity - Sensitive to the presence of near diagonal elements in a GLCM, representing the similarity in gray level between adjacent pixels

* N_g_ = the number of gray levels, g(i,j) = the entry (i,j), μ  = Mean, and $\sigma^{2}\;$ = variance.
